# History of the World Allergy Organization: The World Allergy Organization Congress - XVIII ICACI, Vancouver 2003

**DOI:** 10.1097/WOX.0b013e31822c9a86

**Published:** 2011-08-15

**Authors:** F Estelle R Simons

**Affiliations:** 1 University of Manitoba, Winnipeg, Manitoba, Canada

## Abstract

History of the World Allergy Organization: In 1951, the leaders in allergy from all over the world came together to form the International Association of Allergology and Clinical Immunology (IAACI). For the next 60 years, the allergy world converged at the IAACI triennial meetings, which became biennial in 2003. The international meetings, originally named the International Congress of Allergology and Clinical Immunology (ICACI), are now the World Allergy Congress (WAC) hosted by the World Allergy Organization (WAO). Everyone who has aspired to have worldwide recognition has played a part in IAACI-WAO. The History of the World Allergy Organization traces the global arc of the allergy field over the past 60 years.

The current officers of WAO elected to focus on this rich history, inviting prominent leaders who are interested in being part of this history project to write about their time with IAACI-WAO. This series will be presented in Cancún, México as part of the XXII World Allergy Congress (December 4-8, 2011). Leading up to the Congress in Cancún, the World Allergy Organization Journal is presenting segments of the History as part of the "Notes of Allergy Watchers Series." Please enjoy.

--Michael A. Kaliner, MD

Historian, and Past-President (2006-2007)

World Allergy Organization

## 

In September 2003, the World Allergy Organization (WAO)/International Association of Allergology and Clinical Immunology (IAACI) held a scientifically and financially successful Congress in Vancouver, Canada (Figure 1).

**Figure 1:**
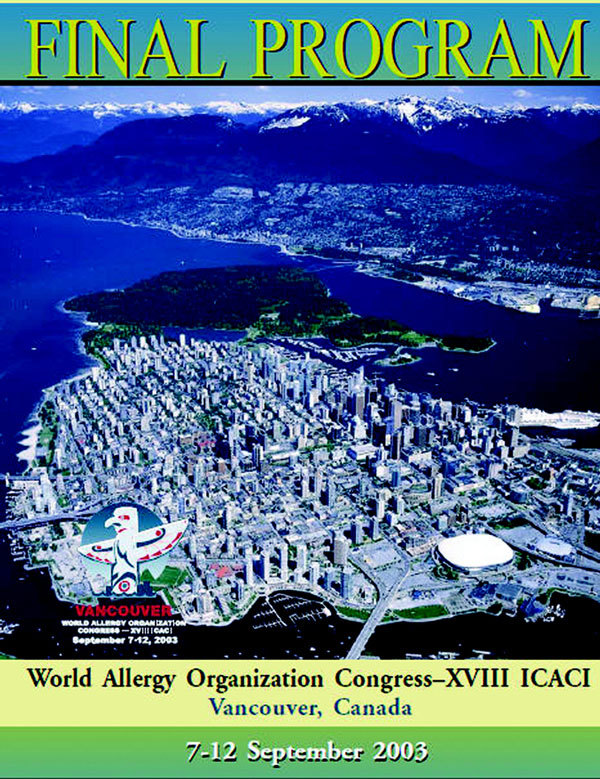
**The cover of the Final Program for the Vancouver Congress featured an aerial view of the city center**.

At this Congress, the name World Allergy Organization was introduced for the first time and used concurrently with the name International Association of Allergology and Clinical Immunology, which it subsequently replaced.

The Congress Organizing Committee was co-chaired by Dr. F. Estelle R. Simons and Dr. Michael A. Kaliner. Committee members were Dr. Allen P. Kaplan, WAO President 2000-2003, and Drs. Carlos Baena-Cagnani, G. Walter Canonica, S.G.O Johansson, and Constance Katelaris.

The Congress was the second one to be held in Canada since the IAACI began in 1951. It took place in one of the world's most attractive and accessible cities. The Vancouver Convention Centre featured magnificent harbor and mountain views from most windows, highlighting the beauty of the area, including a large nearby urban parkland with towering evergreen trees and fabulous beaches.

Dr. Allen Kaplan provided outstanding leadership to the Scientific Program Committee, comprised of Drs. Carlos Baena-Cagnani, Stephen Durham, Takeru Ishikawa, Michael Kaliner, Cas Motala, Johannes Ring, Lanny Rosenwasser, Robert Schellenberg, F. Estelle R. Simons, Daniel Vervloet, and Pakit Vichyanond. The process of developing the Congress was unique in several ways, for example, a needs assessment was conducted, and most of the 270 speakers and moderators in the major sessions representing 6 continents were nominated by their own WAO member societies. More than 800 abstracts were accepted, reflecting cutting-edge allergy and immunology research from laboratories and clinics in 69 nations. The podium and poster sessions included presentations by 30 Congress travel grant winners: 9 young investigators from Europe, 8 from Asia and Australia, 8 from North and South America, and 4 from Africa and the Middle East. Many of these young physicians and scientists are now emerging as leaders in allergy and immunology in their respective countries.

The scientific highlights of the meeting were numerous. **Plenary session **topics and speakers included the following:

The Science of Allergy: Initiating Mechanisms

Drs. William Paul, Andrew Luster, and Kent HayGlass

The Science of Allergy: Effector Mechanisms

Drs. Dean Metcalfe, Marc Rothenberg, and Barry Kay

New Concepts in Asthma

Drs. Stephen Holgate, Richard Martin, and Paul O'Byrne

New Concepts in Allergic Skin Disease

Drs. Allen Kaplan, Thomas Bieber, and Johannes Ring

New Concepts in Allergen Immunotherapy

Drs. Kurt Blaser, Hans-Jorgen Malling, G. Walter Canonica, and Dale Umetsu

**Meet the Professor **sessions featured 16 internationally renowned scientists as chairs and speakers. The topics included the following:

Risks and Benefits of Immunotherapy for Allergic Airway Disease

Chair: Dr. Alain de Weck; Speaker: Dr. Stephen Durham

Leukotrienes in Asthma

Chair: Dr. Stephen Holgate; Speaker: Dr. K. Frank Austen

New Therapeutic Strategies for Allergic Rhinitis

Chair: Dr. Terumasa Miyamoto; Speaker: Dr. Eli Meltzer

Th1/Th2 Polarized Immunity

Chair: Dr. William Busse; Speaker: Dr. Patrick Holt

Cellular and Molecular Aspects of Airway Inflammation

Chair: Dr. Takeshi Fukuda; Speaker: Dr. Barry Kay

Eczema and Dermatitis

Chair: Dr. Albert Oehling; Speaker: Dr. Johannes Ring

Food Allergy

Chair: Dr. Pakit Vichyanond; Speaker: Dr. Hugh Sampson

Asthma: Inflammatory Markers in Induced Sputum

Chair: Dr. Rajendra Prasad; Speaker: Dr. Frederick Hargreave

The 4 **Debates of the Day **, featuring timely topics and 8 well-known professors, proved to be a novel and popular attraction:

*Immunotherapy for Asthma? *--Dr. Anthony Frew versus Dr. N. Franklin Adkinson

*Kill the Cat or Buy One? *--Dr. Adnan Custovic versus Dr. Thomas Platts-Mills

*Is the Eosinophil a Player or an Interested Bystander in Asthma? *--Dr. Redwan Moqbel versus Dr. Robert Schleimer *Chronic Sinusitis: Infection or Allergy? *--Dr. Claus Bachert versus Dr. Michael Kaliner

More than 30 symposia highlighted the most important allergy/immunology topics of the early 21st century. These included the impact of upper airway allergic inflammation on asthma (WAO Allergy Forum) and advances in occupational allergy (the Canadian Society of Allergy and Clinical Immunology symposium). Novel interventions for asthma were featured in symposia on dual inhaled glucocorticoid/longacting beta-agonist therapy, and on anti-IgE therapy. Other symposia focused on the genetics of allergic disease, new trigger factors for allergic disease, interventions to halt the atopic march, and prevention of anaphylaxis recurrences in the community. Additional symposia highlighted the latest information on DNA vaccines, peptide vaccines, and cytokine/chemokine antagonists.

The Local Arrangements Subcommittee was co-chaired by Drs. Robert Schellenberg and Donald Stark. Subcommittee members included Drs. Michael Mandl, Ross Chang, Alexander Ferguson, John Dean, H.C. George Wong, Liliane Gendreau-Reid, and Parminder Singh; Gloria Schellenberg, Patricia Stark, Jo-Anne Gillespie, Leslie Chang, Joyce Ferguson, and Narinder Chauhan.

The social activities highlighted the geographical, historical, and cultural significance of the area. The Opening Ceremony and Welcome Reception focused on the proud heritage and seafaring traditions of the local First Nations peoples, told through music, story, dance, and feasting. For the Congress Banquet, a Northern Lights gala dinner and dance, the *aurora borealis *was recreated along with a magical backdrop of ice sculptures. At the informal All-Congress WAO Western barbeque held at B.C. Place Stadium, delegates enjoyed authentic Western cuisine and danced the night away. At this event, some attendees wore blue jeans for the first time ever, others panned for gold, and the Royal Canadian Mounted Police made a surprise visit.

Complimentary sightseeing tours were organized for Congress registrants to orient them to the spectacular city of Vancouver, and optional tours were organized to the world-renowned Museum of Anthropology at the University of British Columbia and other Vancouver landmarks.

Some delegates traveled the sea-to-sky highway to Whistler Mountain for hiking, gondola rides, canoe rides, and floatplane tours of the glaciers. Others visited Victoria, the beautiful capital city of the province of British Columbia, and a few braved the open Pacific Ocean to go on whale-watching expeditions. Participants were amazed at the ease of access from the Vancouver and Victoria urban areas to the nearby vast and magnificent Canadian wilderness. A number of fortunate delegates experienced one of the world's great panoramic railway journeys, traveling on the Rocky Mountaineer from Vancouver through scenic British Columbia to Banff National Park.

Seven years in international planning... 5 wonderful days in our lives.... a lifetime of memories... This sums up the 2003 World Allergy Congress in beautiful Vancouver, Canada.

